# Bias in HD-ISS staging introduced by the FreeSurfer cross-sectional stream: Insights from the Huntington's Disease Young Adult Study (HD-YAS)

**DOI:** 10.1177/18796397251366900

**Published:** 2025-08-13

**Authors:** Harry Knights, Annabelle Coleman, Mena Farag, Michela Leocadi, Michael Murphy, Kate Fayer, Olivia Thackeray, Douglas Langbehn, Nicola Hobbs, Sarah J Tabrizi, Rachael I Scahill, the HD-YAS investigators

**Affiliations:** 1 61554 Huntington's Disease Centre, Department of Neurodegenerative Disease, UCL Queen Square Institute of Neurology, University College London, London, UK; 2 Department of Psychiatry and Biostatistics, Carver College of Medicine and College of Public Health, 4083 University of Iowa, Iowa City, IA, USA

**Keywords:** huntington's disease, FreeSurfer, MRI, segmentation, caudate, putamen, HD-ISS

## Abstract

Huntington's Disease Integrated Staging System (HD-ISS) stages are likely inclusion criteria in future clinical trials. Stage 1 volumetric cut-offs were derived using the FreeSurfer longitudinal stream (LG). However, trials will require cross-sectional stream (CS) application with one MRI. Volumetric outputs are not robust to software type or version. T1-weighted images from 88 participants with MRIs from baseline and follow-up HD-YAS visits were segmented using both streams. CS calculated smaller caudate and putamen volumes adjusted for total intracranial volume, with greater reduction for larger volumes, shifting towards HD-ISS stage 1. CS-specific cut-offs need to be established before application to clinical trials.

## Introduction

Huntington's disease (HD) is a devastating progressive neurodegenerative disorder for which there remains no disease-modifying therapy. Inherited CAG repeat expansion undergoes further somatic expansion towards a critical length in vulnerable cell types, particularly the medium-spiny neurons in the striatum,^1^ causing neuronal damage, dysfunction, and death.^2^ This results in early atrophy within the striatum^3^ which extends over time to other subcortical and cortical regions.^4,5^

The HD Young Adult Study (HD-YAS) is a deeply phenotyped far-from-onset observational study of closely matched HD gene expanded (HDGE) and control groups, assessed longitudinally at two visits (V1 and V2) approximately 4.5 years apart.^3^

The Huntington's Disease Integrated Staging System (HD-ISS) re-defined pre-manifest HD as stage 0 (without detectable biomarkers of pathophysiology) and 1 (caudate and/or putamen atrophy below the 5^th^ percentile in healthy controls).^6^

We now enter a pivotal period for HD research, with multiple ongoing clinical trials of disease-modifying therapies.^7^ Secondary prevention trials aim to target underlying disease pathobiology before significant neurodegeneration, with the goal of preserving the functional integrity of remaining striatal circuitry. Certain experimental therapies are also aiming to interfere with very early pathobiological mechanisms, including somatic expansion.^8,9^ HD-ISS stages 0 and 1 are therefore possible inclusion criteria in future secondary prevention trials.

The HD-ISS used the FreeSurfer (FS) longitudinal stream (LG) to define stage 1 cut-offs, with the aim of reducing within-subject variability.^10^ However, clinical trials will likely require application to participants with a single MRI, necessitating cross-sectional stream (CS) processing. MRI-derived volumetric outputs are not robust to pipeline^11^ or software version,^12^ and the impact of stream on volumes and staging remains unknown.

This study will explore the relationships and differences between caudate, putamen, and total intracranial volumes (TICV) derived from FS CS and LG in HD-YAS.

## Methods

### Study participants

HD-YAS participants were originally recruited from Enroll-HD (https://www.enroll-hd.org/), regional genetic and HD centers, the Huntington's Disease Association (https://www.hda.org.uk/), and the Huntington's Disease Youth Organisation (https://hdyo.org/). Participants were aged 18–40 inclusive and were excluded if they had a history of drug and/or alcohol abuse, significant co-morbidity, or contraindications to MRI. The HDGE group were required to have no clinical diagnostic motor features of HD (Unified Huntington's Disease Rating Scale [UHDRS] Diagnostic Confidence Level < 4), CAG repeat expansion length ≥ 40, and Disease Burden Scores (DBS)^13^ ≤ 240. Controls were at-risk gene-negative family members (CAG < 36), genetically unrelated family members, and members of the wider HD community. HDGE and control groups were matched for age, sex, and education using means and variances. Participants were enrolled from August 2017 to April 2019 for visit 1 (V1), and April 2022 to January 2024 for visit 2 (V2). 88 participants (54 HDGE individuals and 34 controls) with neuroimaging performed at both V1 and V2 were included in this study.

### MRI acquisition

All MRIs were acquired using the same research-dedicated 3-Tesla Prisma scanner (Siemens Healthcare, Erlangen, Germany). T1-weighted images were acquired using a 3D Magnetization Prepared Rapid Gradient Echo (MPRAGE) with the following parameters: repetition time = 2530 ms; time to echo = 3.34 ms; inversion time = 1100 ms; flip angle = 7◦; field of view = 256 × 256 × 176 mm^3^; and resolution = 1.0 × 1.0 × 1.0 mm^3^.

HD-YAS MRI scans are high quality and well-standardized due to the use of the same research dedicated MRI scanner, imaging protocols optimized for grey/white segmentation, experienced radiographers, and minimal motion artefacts in the far-from-onset HDGE group.

### FreeSurfer

All segmentations were run on FS version 6.0.1 to mirror the methodology used to define staging cut-offs^6^ and because segmentations are not robust to software version due to modifications to segmentation algorithms.^12^ All segmentations were run on the same operating system, since this has also been shown to influence segmentation outputs.^14,15^ The explanation for this is complex and arises from a combination of factors, including how different operating systems handle floating-point numbers (i.e., rounding errors), how they order calculations, and how FS is compiled for each system (how it is integrated with the operating system).^15^

For CS, segmentations were generated using recon-all.^16–18^ In brief, T1-weighted images undergo skull-stripping, an affine transform to MNI305 space,^19^ intensity homogenization, and a non-linear transform. Probability distributions for voxel location and intensity are derived from the Talairach atlas.^20^ Volume is calculated as the number of voxels of known size (usually 1 mm^3^) within the region-of-interest. TICV is inferred from the scaling factor required for the affine transformation to Talairach space.^21^

For LG, CS segmentations from V1 and V2 were combined to create an unbiased within-subject template using recon-base.^22,23^ Image processing is then initialized using common information from the within-subject template (avoiding interpolation asymmetry), and each time-point is processed individually (without temporal regularization) using the recon-long command.^10^ LG applies a fixed affine transformation across time-points, therefore deriving the same TICV at all time-points.

A flow diagram containing the processing steps for both streams is displayed in Supplemental Figure 1.

### Quality control

Volumes may remain within the normal range despite inaccurate segmentations and therefore segmentations must be reviewed visually.^24^ CS and LG segmentations for V1 and V2 were quality controlled by a single investigator (HK) blinded to disease status and volume. Segmentations were considered to be ‘pass’ or ‘fail’ based on whether the segmented boundaries were significantly outside of the visible boundary. No segmentations were identified as gross failures for either stream. Manual editing of segmentations was not performed. TICV could not be quality controlled since it is based on the affine transform to the Talairach atlas and no region is generated, consistent with HD-ISS methodology.^6^

### Statistical analyses

Differences between volumes were explored using Bland-Altman analysis.^25^ A scatter plot was created in which the y-axis shows the difference between two volumes (A–B), and the x-axis shows the mean between two volumes ([A + B]/2). Systematic bias was described using the mean difference and 95% limits of agreement. Proportional bias was explored through linear regression analysis.^26^ Similarities between CS and LG volumes were also assessed using intraclass correlation coefficient (ICC). A two-tailed p-value below 0.05 was considered statistically significant. All statistics were performed using Stata v17.0.

## Results

Baseline demographics for participants are displayed in Supplemental Table 1.

All ICC values between CS and LG volumes were > 0.97 and were highly significant at p < 0.0005 (Supplemental Table 2).

**Figure 1.**
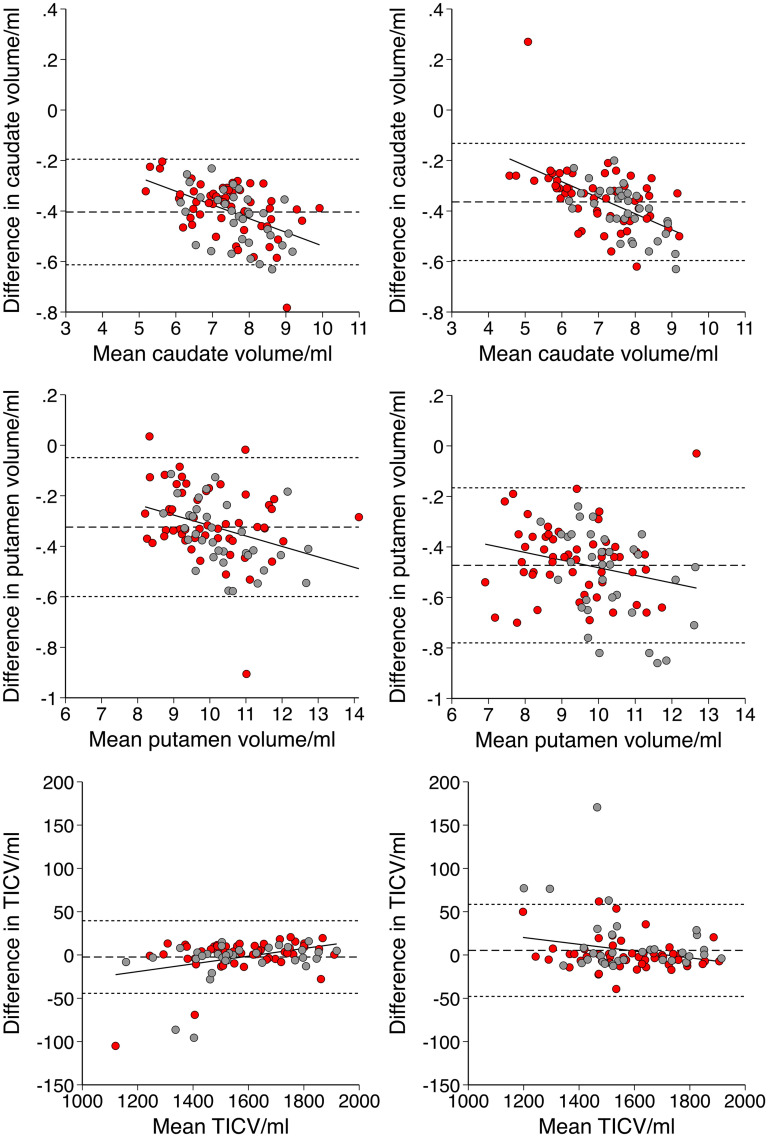

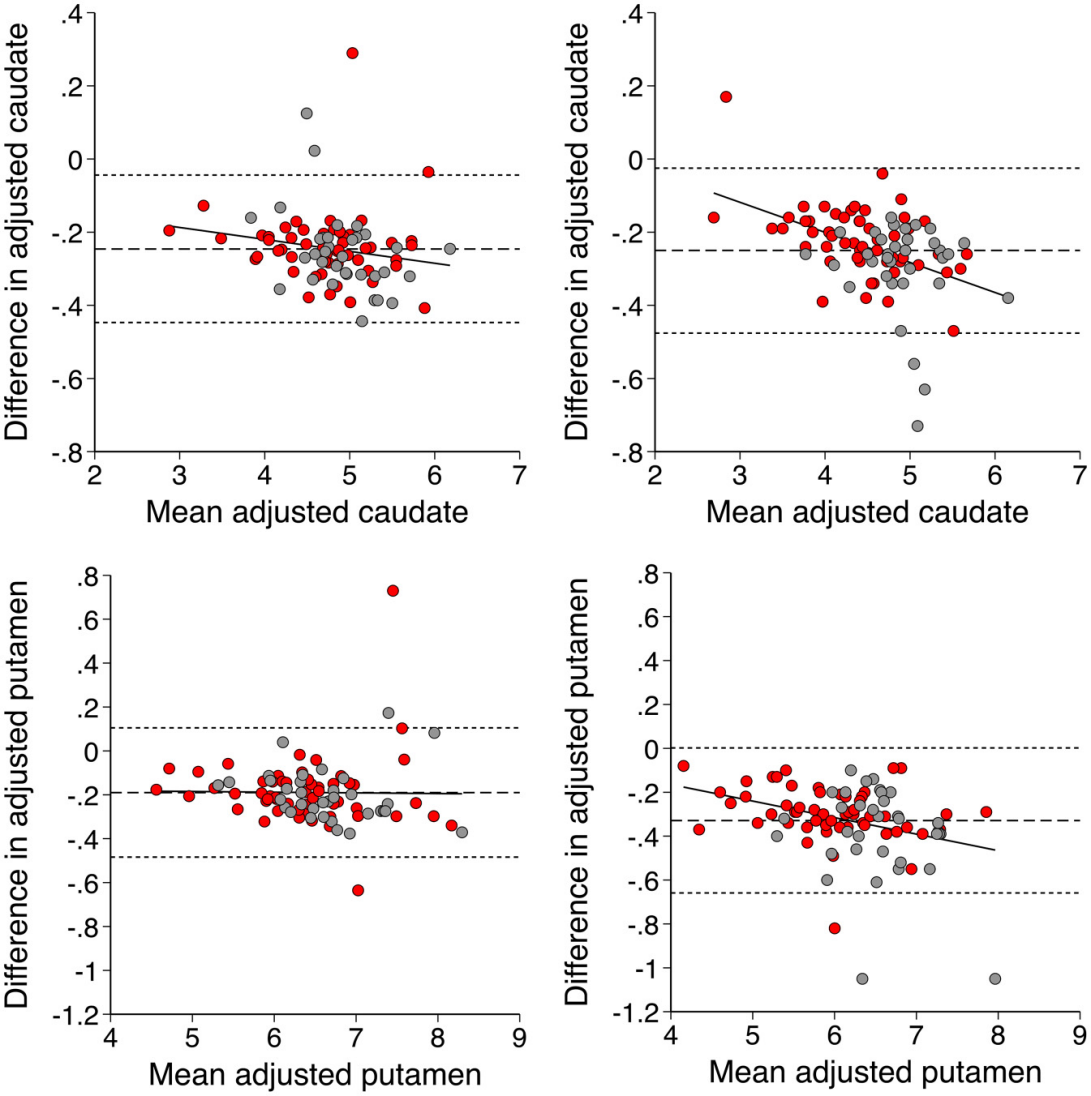
Bland-Altman plots with differences represented as cross-sectional (CS) – longitudinal (LG) stream volumes

Differences between volumes were described using Bland-Altman analysis (plots displayed in Figure 1 and systematic differences displayed in Supplemental Table 3). Compared to LG, CS calculated: i) smaller raw caudate (−5.5% V1, −5.1% V2, both p < 0.00005) and putamen volumes (−3.2% V1, −5.0% V2, both p < 0.00005), with a bias towards greater reduction for larger volumes and ii) similar TICV (−0.2% V1, + 0.3% V2, both p > 0.05). This resulted in smaller adjusted caudate (−5.3% V1, −5.6% V2, both p < 0.00005, without proportional bias) and putamen (−3.0% V1, −5.5% V2, both p < 0.00005, with greater reduction for larger volumes).

Total intracranial volume (TICV). Adjusted caudate and putamen are expressed as (raw volume/TICV)*1000. Left-sided plots are for visit 1 (V1) and right-sided plots are for visit 2 (V2). Data points are labelled as the HDGE (red) and control (grey) groups. Linear regression (solid), mean difference (dash), 95% limits of agreement (dots). Linear regression equations are depicted in Supplemental Table 3.

**Figure 2.**
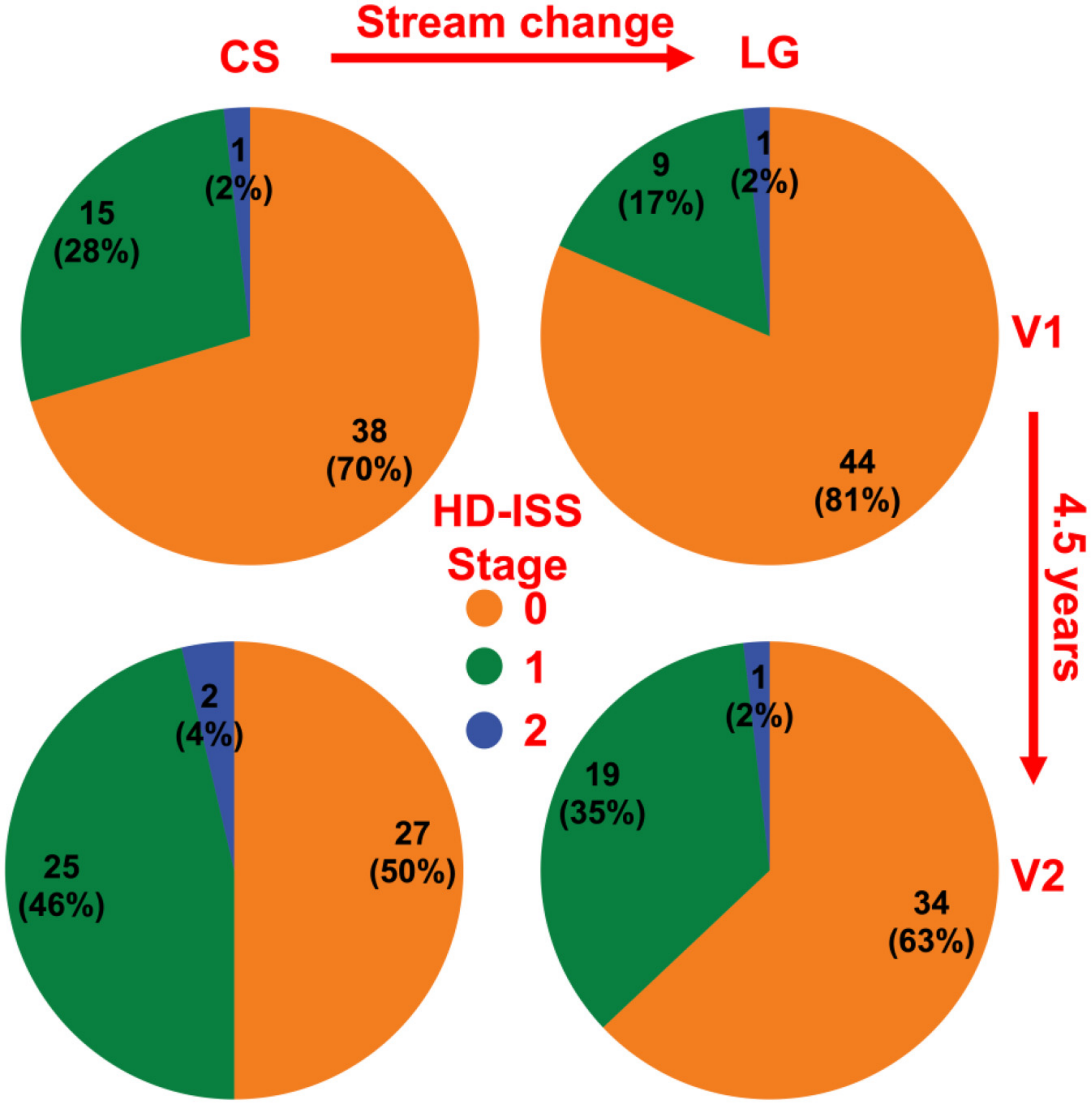
HD-ISS staging according to the FreeSurfer cross-sectional (CS) and longitudinal (LG) streams

These volumetric differences impacted staging, with CS shifting the HDGE group from stage 0 to 1, moving from 9/54 to 15/54 at V1 and 19/54 to 25/54 at V2 (Figure 2).

Staging for the HDGE group (n = 54) with brain MRIs at visit 1 (V1) and visit 2 (V2) are displayed. Moving from left to right changes from CS to LG. Moving from top to bottom changes from V1 to V2.

These findings suggest that CS estimates larger adjusted caudate and putamen volumes which are less likely to reach the stage 1 threshold, according to the existing longitudinally derived cut-offs.

## Discussion

This study adds to the growing body of evidence describing the meaningful impact of subtle pipeline differences on striatal segmentations, volumetric outputs, and HD-ISS staging in HDGE individuals. The use of HD-ISS staging as inclusion criteria in interventional trials will likely require application to HDGE individuals with a single MRI brain scan, precluding the use of LG.

Previous studies comparing CS and LG caudate and putamen segmentations are uncommon and have focused on reliability, showing improved test-retest reproducibility.^10,27,28^ This is useful for assessing sensitivity to detect subtle change over time, which impacts sample sizes and follow-up periods in interventional trials. However, the impact of stream of staging requires an exploration of measurement bias.

This study has shown that caudate, putamen, and total intracranial volumes varied greatly between CS and LG. The combined effect was that CS estimated smaller adjusted caudate and putamen volumes, with greater effect on larger volumes. This shifted the HDGE group towards stage 1 (Figure 2). In particular, the use of CS at V1, and LG at V2, as might be assumed to be a reasonable methodology to maximize the accuracy of individual segmentations with growing available data, functioned to substantially reduce the staging progression between time-points.

It should be noted, however, that LG may be less suitable for estimating volumes when there is substantial atrophy over time. This limitation arises from a combination of factors: template bias (where a later atrophic scan can distort the segmentation of an earlier healthy scan); registration errors; and non-linear degeneration.^10^ However, with minimal atrophy during the transition from HD-ISS stage 0 and 1, LG remains appropriate for use in this context.

The mechanistic explanation for these differences is challenging to define. LS applies the same affine transform to all time-points, deriving the same TICV, which is approximately the average of the two cross-sectional TICVs (Supplemental Figure 2), explaining the non-significant difference in TICV. Differences in caudate and putamen segmentation likely relate to the creation of a within-subject median template image to initialize the segmentation.

Overall, applying the HD-ISS to interventional trials requires staging with CS. This calculates larger adjusted caudate and putamen volumes than LG, which was used to generate stage 1 cut-offs, shifting from stage 0 to 1 across time-points. The HD-ISS must urgently define cut-offs derived from CS and make them publicly available before widespread application in clinical trials.

## Supplemental Material

sj-docx-1-hun-10.1177_18796397251366900 - Supplemental material for Bias in HD-ISS staging introduced by the FreeSurfer cross-sectional stream: Insights from the Huntington's Disease Young Adult Study (HD-YAS) Supplemental material, sj-docx-1-hun-10.1177_18796397251366900 for Bias in HD-ISS staging introduced by the FreeSurfer cross-sectional stream: Insights from the Huntington's Disease Young Adult Study (HD-YAS) by Harry Knights, Annabelle Coleman, Mena Farag, Michela Leocadi, Michael Murphy, Kate Fayer, Olivia Thackeray, Douglas Langbehn, Nicola Hobbs, Sarah J Tabrizi, Rachael I Scahill and in Journal of Huntington's Disease
