# Supplementary material for: Bias in HD-ISS staging introduced by the FreeSurfer cross-sectional stream: Insights from the Huntington's Disease Young Adult Study (HD-YAS)
Source: J Huntingtons Dis. 2025 Aug 13;14(4):396–402. doi: 10.1177/18796397251366900 (PMC12602721; doi:10.1177/18796397251366900)
Supplement: sj-docx-1-hun-10.1177_18796397251366900 - Supplemental material for Bias in HD-ISS staging introduced by the FreeSurfer cross-sectional stream: Insights from the Huntington's Disease Young Adult Study (HD-YAS) [file sj-docx-1-hun-10.1177_18796397251366900.docx]

**Supplemental Material**

**Bias in HD-ISS staging introduced by the FreeSurfer cross-sectional stream: Insights from the Huntington’s Disease Young Adult Study (HD-YAS)**

**Supplemental Table 1.** Demographics and clinical characteristics

|  | **V1** | **V2** |
| --- | --- | --- |
| **All** | N=88 | |
| **Male** | 41 (46.6%) | |
| **Age (years)** | 29.9 (25.0 – 34.9) | 34.7 (30.0 – 39.6) |
| **Education (ISCED)** | 5 (3 – 5) | 5 (4.5 – 5) |
| **Controls** | n=34 (38.6%) | |
| **Male** | 14 (41.2%) | |
| **Age (years)** | 29.0 (25.5 – 36.0) | 33.9 (30.8 – 40.9) |
| **Education (ISCED)** | 5 (5 – 5) | 5 (5 – 5) |
| **HDGE** | n=54 (61.4%) | |
| **Male** | 27 (50.0%) | |
| **Age (years)** | 31.1 (24.4 – 34.8) | 35.6 (29.6 – 38.7) |
| **Education (ISCED)** | 5 (3 – 5) | 5 (4 – 5) |
| **CAG length** | 42 (41 – 43) | |
| **DBS** | 196 (39) | 228 (44) |
| **TMS** | 0 (0 – 1) | 0 (0 – 2) |
| **SDMT** | 58.9 (9.3) | 57.7 (9.8) |

Demographics and clinical characteristics of the whole cohort, control, and HDGE groups at visits 1 (V1) and 2 (V2). ISCED: International Standard Classification of Education; DBS: Disease Burden Score; TMS: Total Motor Score; SDMT: Symbol Digit Modalities Test

**Supplemental Table 2.** Intraclass correlation coefficients (ICCs) for segmentation volumes from the cross-sectional (CS) and longitudinal (LG) streams

|  |  | **ICC** | ***p*** |
| --- | --- | --- | --- |
| **Raw caudate** | **V1** | 0.99 | **<0.0005** |
|  | **V2** | 0.99 | **<0.0005** |
| **Raw putamen** | **V1** | 0.99 | **<0.0005** |
|  | **V2** | 0.99 | **<0.0005** |
| **TICV** | **V1** | 0.99 | **<0.0005** |
|  | **V2** | 0.99 | **<0.0005** |
| **Adjusted caudate** | **V1** | 0.98 | **<0.0005** |
|  | **V2** | 0.98 | **<0.0005** |
| **Adjusted putamen** | **V1** | 0.98 | **<0.0005** |
|  | **V2** | 0.97 | **<0.0005** |

ICC values are displayed for visits 1 (V1) and 2 (V2). TICV: total intracranial volume

**Supplemental Table 3.** Systematic differences in segmentation volumes from the cross-sectional (CS) and longitudinal (LG) streams

|  | | **LG** | **CS** | **Mean difference** | **%**  **difference** | **LOA** | ***p*** |
| --- | --- | --- | --- | --- | --- | --- | --- |
| **Raw caudate (ml)** | **V1** | 7.73 (1.01) | 7.32 (0.96) | -0.4 | -5.52 | -2.28 to 1.47 | **<0.00005** |
|  | **V2** | 7.44 (1.1) | 7.07 (1.03) | -0.36 | -5.14 | -2.39 to 1.66 | **<0.00005** |
| **Raw putamen (ml)** | **V1** | 10.31 (1.17) | 9.98 (1.12) | -0.32 | -3.24 | -2.52 to 1.87 | **<0.00005** |
|  | **V2** | 9.95 (1.28) | 9.48 (1.24) | -0.47 | -4.99 | -2.91 to 1.96 | **<0.00005** |
| **TICV (ml)** | **V1** | 1578 (173) | 1576 (180) | -2.41 | -0.15 | -356 to 351 | 0.29 |
|  | **V2** | 1578 (173) | 1583 (165) | 5.3 | 0.34 | -320 to 331 | 0.07 |
| **Adjusted caudate** | **V1** | 4.92 (0.58) | 4.67 (0.56) | -0.25 | -5.26 | -1.35 to 0.86 | **<0.00005** |
|  | **V2** | 4.73 (0.64) | 4.48 (0.59) | -0.25 | -5.59 | -1.4 to 0.9 | **<0.00005** |
| **Adjusted putamen** | **V1** | 6.57 (0.73) | 6.38 (0.73) | -0.19 | -2.97 | -1.62 to 1.24 | **<0.00005** |
|  | **V2** | 6.34 (0.77) | 6.01 (0.72) | -0.33 | -5.47 | -1.73 to 1.08 | **<0.00005** |

Systematic differences are displayed for visits 1 (V1) and 2 (V2). Total intracranial volume (TICV) and limits of agreement (LOA). Adjusted caudate and putamen are expressed as (raw volume/TICV)*1000.

**Supplemental Figure 1.** FreeSurfer processing streams

**
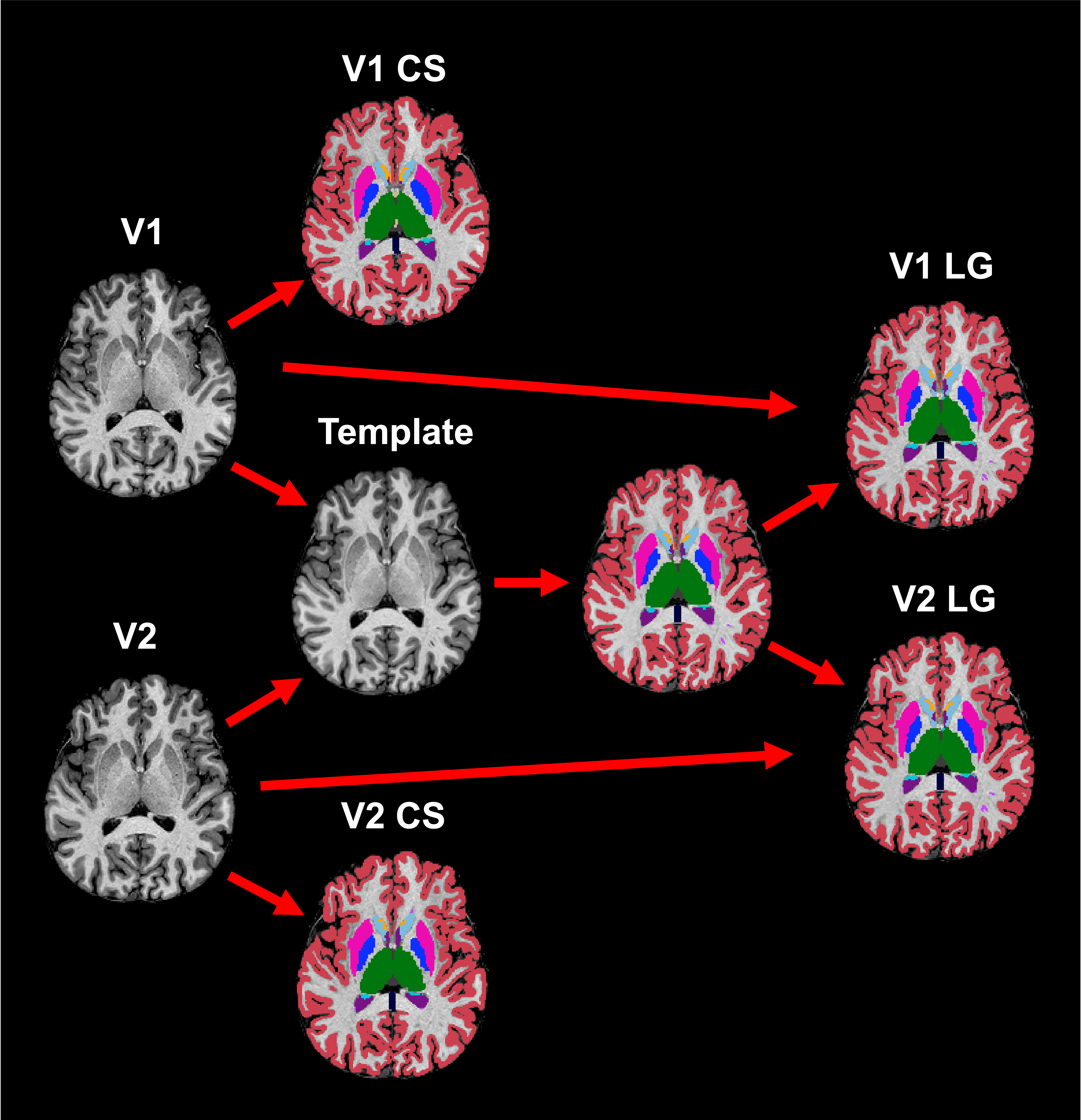
**

The FreeSurfer cross-sectional stream (CS) segments each time-point individually, while the longitudinal stream (LG) creates and segments a within-subject spatially unbiased template, which is then used to initialize the segmentation. Visit 1 (V1) and 2 (V2).

**Supplemental Figure 2.** Mean total intracranial volume (TICV) from cross-sectional (CS) processing of visit 1 (V1) and 2 (V2) against TICV from the longitudinal (LG) stream
